# *In situ* vaccination for the treatment of localized cancer and the prevention of metastatic disease

**DOI:** 10.3389/fimmu.2026.1862956

**Published:** 2026-09-22

**Authors:** Sangbeom Lee, Moonhae Kim, Yian Wang, Bryon Johnson, Ming You

**Affiliations:** 1 Center for Cancer Prevention, Houston Methodist Cancer Center, Houston Methodist Research Institute, Houston, TX, United States; 2 Department of Kinesiology, Rice University, Houston, TX, United States; 3 Division of Hematology and Oncology, Department of Medicine, Medical College of Wisconsin, Milwaukee, WI, United States

**Keywords:** biomaterials and drug delivery, dendritic cell cross-priming, immunogenic cell death, *in situ* vaccination, intratumoral immunotherapy, radiotherapy fractionation

## Abstract

*In situ* vaccination (ISV) is a local tumor-directed immunotherapy strategy that uses a treated tumor as an endogenous, patient-specific source of tumor antigens and a site of immune activation, with the goal of converting local treatment into systemic and durable antitumor immunity. Depending on the modality, ISV induces immunogenic cell death, other forms of tumor-cell stress or death, or local innate immune activation, thereby increasing antigen availability, promoting type I interferon signaling, and supporting dendritic cell–mediated cross-priming of tumor-reactive T cells capable of mediating both local and systemic antitumor immunity, including regression of untreated lesions. Across immunocompetent preclinical models, ISV can generate durable local tumor control, abscopal responses, and long-term immune memory, particularly when combined with modalities that amplify antigen release or inflammatory signaling. Early clinical studies employing intratumoral innate immune agonists, radiotherapy-based regimens, cytokines, and oncolytic viruses demonstrate clinical feasibility, favorable safety profiles, and emerging evidence of systemic efficacy in selected tumor types. In this review, we discuss the immunologic principles underlying ISV and organize the field according to major triggering modalities, including radiotherapy, ablation, and light-based therapies, selected chemotherapies, innate immune agonists, cytokines, and oncolytic viruses. We further highlight emerging intratumoral delivery platforms, such as lipid nanoparticles, injectable hydrogels, and biodegradable scaffolds, that enable precise spatial and temporal control of immune activation within the tumor microenvironment. Finally, we review the evidence that locally initiated antitumor immunity can extend beyond the treated lesion to control untreated lesions and metastatic disease, and discuss the challenges associated with achieving durable systemic efficacy and preventing recurrence.

## Graphical Abstract

**Figure:**
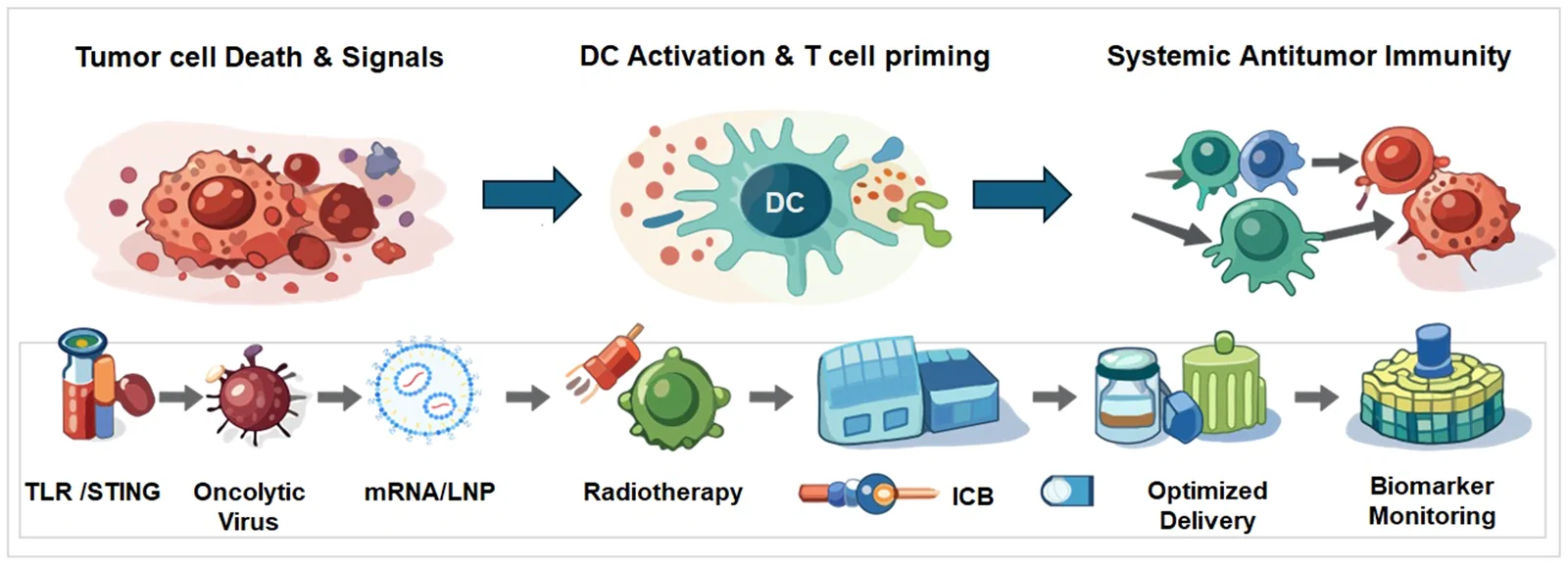
*In situ* vaccination (ISV) uses a locally treated tumor as a source of tumor antigens and immune activation. Tumor-cell stress or death, together with local innate immune signaling, promotes dendritic-cell activation, cDC1-mediated antigen cross-presentation, and tumor-reactive T-cell responses. Effector and memory T cells may then reach untreated lesions and contribute to systemic and durable antitumor immunity. Major ISV approaches include radiotherapy, tumor ablation, oncolytic viruses, intratumoral innate immune agonists, nucleic-acid platforms, and selected immunogenic cell death–inducing treatments. Their efficacy may be improved through rational combinations, controlled delivery, and biomarker-guided patient selection.

## Introduction

Cancer immunotherapy has transformed modern oncology, producing durable remissions in malignancies once considered uniformly lethal. Immune checkpoint blockade (ICB) targeting programmed cell death protein 1 (PD-1), programmed death ligand 1 (PD-L1), and cytotoxic T-lymphocyte–associated antigen 4 (CTLA-4) has improved long-term survival across multiple cancers. Engineered cellular therapies, including FDA-approved chimeric antigen receptor (CAR) T cells for B-cell malignancies and tumor-infiltrating lymphocyte (TIL), and CAR natural killer (CAR-NK) approaches for solid tumors, have further expanded the field. Therapeutic cancer vaccines based on shared antigens or personalized neoantigens delivered by DNA, RNA, or viral vectors have also advanced toward clinical translation. However, the benefits of immunotherapy in solid tumors remain heterogeneous because many tumors are poorly immunogenic, highly heterogeneous, and immunosuppressive, thereby impairing antigen presentation, T-cell priming, immune cell trafficking, and effector function. In addition, several approaches face logistical, manufacturing, and scalability barriers that limit broad accessibility and reproducibility (1–3).

The concept of stimulating antitumor immunity directly at the tumor site predates modern immunology. In the late nineteenth century, William B. Coley reported tumor regressions after intratumoral injection of mixed bacterial lysates (“Coley’s toxins”), providing early evidence that localized immune activation could lead to systemic tumor control (4). In the modern era, this principle evolved into *in situ* vaccination (ISV), which uses a locally treated tumor as a source of endogenous tumor antigens and a site of immune activation (5).

ISV aims to convert an accessible tumor lesion into its own vaccine source. In many ISV approaches, local tumor perturbation induces immunogenic cell death (ICD), releasing or exposing endogenous tumor antigens together with damage-associated molecular patterns (DAMPs) such as calreticulin (CRT), extracellular ATP, high-mobility group box 1 (HMGB1), and heat-shock proteins. These signals engage pattern-recognition receptors (PRRs) and activate type-I interferon (IFN-I) programs that are essential for productive antigen presentation (6). Batf3-dependent type 1 conventional dendritic cells (cDC1s) then capture tumor antigens, migrate to tumor-draining lymph nodes, and support the priming of tumor-reactive T cells, promoting effector expansion, epitope spreading, and systemic immune surveillance (7–9). Regression of an untreated tumor outside the local treatment field, commonly termed an abscopal response, was initially described in the context of RT and may be enhanced by immune modulation, particularly ICB (10–12). More broadly, ISV depends not only on antigen release but also on the quality, magnitude, and timing of innate immune activation. A major benefit of ISV is its capacity to convert focal treatment into systemic antitumor immunity. Different ISV modalities engage this immunologic cascade through distinct mechanisms. Radiotherapy, ablation, light-based therapies, and selected chemotherapies primarily increase tumor-antigen release and inflammatory signaling, whereas intratumoral innate immune agonists and cytokines provide additional immune activation within the tumor microenvironment (13–19). Consistent with these principles, preclinical studies have shown that ISV can induce regression of treated tumors, control of untreated lesions, and durable immune memory.

Previous reviews have summarized the biological basis and major therapeutic approaches of ISV and intratumoral immunotherapy (20, 57). In this review, we focus on how local ISV treatment can generate systemic and durable antitumor responses beyond the treated lesion. We compare quantitative preclinical and clinical evidence across major ISV modalities, with particular attention to responses in untreated lesions, metastatic control, recurrence, and factors that limit reproducibility and clinical translation.

## Mechanisms of *in situ* vaccination

ISV is an immunotherapeutic strategy in which the tumor itself is converted into a vaccine, eliminating the need for ex vivo antigen isolation, formulation, and delivery. By inducing ICD or other forms of tumor-cell stress or death, or by promoting local innate immune activation directly at the tumor site, ISV allows the immune system to sample the complete repertoire of tumor antigens, including subclonal epitopes. This localized immune priming can generate systemic cytotoxic T-cell responses capable of eliminating malignant cells at both treated and untreated metastatic sites. Its efficacy depends on the coordinated execution of three interdependent processes: (i) generation or exposure of tumor antigens in an inflammatory context; (ii) uptake, processing, and presentation of these antigens by professional antigen-presenting cells (APCs); and (iii) priming and expansion of tumor-reactive T cells (20).

ISV employs tumor antigens, which are peptides or proteins expressed by malignant cells and recognized by the host immune system. These are broadly classified into tumor-associated antigens (TAAs) and tumor-specific antigens (TSAs). TAAs derive from self-antigens aberrantly expressed in cancer, including overexpressed or amplified proteins such as HER2/neu in breast cancer (21), developmentally re-expressed proteins such as alpha-fetoprotein in hepatocellular carcinoma (22), and cancer-testis antigens such as MAGE family members in melanoma (23, 24). Because TAAs originate from self, immune responses against them are often limited by central and peripheral tolerance. In contrast, TSAs arise from tumor-specific genetic alterations that generate neoantigens including point mutations such as TP53 missense mutations (25) and chromosomal rearrangements generating fusion proteins such as BCR–ABL (26). Because TSAs are not subject to central tolerance, they are highly immunogenic and attractive therapeutic targets. Depending on the ISV modality, tumor antigens become available through apoptosis, necrosis, necroptosis, viral lysis, or other forms of tumor-cell stress and are taken up by dendritic cells and other APCs through phagocytosis or receptor-mediated uptake. A major advantage of ISV is that it makes both TAAs and TSAs available for uptake and presentation *in situ* without requiring prior knowledge of antigen identity.

In ISV approaches that induce tumor-cell death, ICD can serve as an initiating event that converts antigen release into productive immune priming. Unlike non-immunogenic apoptosis, ICD couples tumor cell death to the emission of danger signals that activate innate immunity. During ICD, dying tumor cells release tumor antigens together with damage-associated molecular patterns (DAMPs), providing both signal 1 (antigen) and signal 2 (danger) for adaptive immune activation. Endoplasmic reticulum–resident proteins such as CRT and heat shock proteins (HSPs) translocate to the tumor cell surface, where CRT serves as an “eat-me” signal promoting phagocytosis by APCs, while HSPs support antigen chaperoning, cross-presentation, and NK cell activation (27). Extracellular ATP and high-mobility group box 1 (HMGB1) further recruit and activate APCs at the site of cell death. However, ATP and HMGB1 alone do not define ICD, as they may also be released during non-immunogenic death. Rather, ICD reflects the coordinated emission of multiple signals in the proper temporal and spatial context to license effective antigen presentation (28).

DAMPs released during ICD engage PRRs, including TLRs and NOD-like receptors, on APCs, triggering maturation, cytokine secretion, and enhanced antigen processing. Among APC subsets, dendritic cells (DCs) are central to ISV because they bridge innate and adaptive immunity. DCs express both major histocompatibility complex (MHC) class I and class II molecules, enabling presentation of intracellular antigens to CD8^+^ cytotoxic T lymphocytes (CTLs) and extracellular antigens to CD4^+^ helper T cells. Their capacity to cross-present extracellular tumor antigens on MHC class I is especially critical for robust CTL responses against solid tumors (29). Effective T-cell activation requires not only peptide–MHC recognition but also co-stimulatory signals, such as CD28 engagement with CD80/CD86 on APCs, and an appropriate pro-inflammatory cytokine milieu within tumor-draining lymph nodes (30). Without sufficient co-stimulation and inflammation, antigen recognition instead leads to T-cell anergy, deletion, or tolerance (31). Thus, effective ISV requires coordination of antigen availability with inflammatory licensing signals.

Once primed, effector T cells exit tumor-draining lymph nodes and disseminate systemically through blood and lymphatics. These cells can recognize and eliminate tumor cells at distant untreated sites, a phenomenon known as the abscopal effect (32). In this way, localized ISV functions as an endogenous, patient-specific vaccine capable of generating body-wide antitumor immunity. CD8^+^ CTLs are the principal mediators of distant tumor killing through perforin and granzyme-dependent mechanisms, while CD4^+^ helper T cells sustain this response through cytokine production, APC licensing, and support of NK cells and macrophages. Together, these responses transform focal tumor destruction into systemic immune surveillance. Although the abscopal effect was first described with RT, abscopal responses may be enhanced when ICD modalities are combined with other immunomodulatory agents (12). The capacity to generate immune memory distinguishes ISV from purely cytotoxic local therapies and underlies its long-term therapeutic potential. The relative contribution of antigen release, innate immune activation, dendritic-cell cross-presentation, and T-cell priming differs among ISV approaches and is discussed in the modality-specific sections below.

## *In situ* vaccination strategies and modalities

ISV is not defined by a single agent, but encompasses diverse approaches that share a common immunologic objective to induce immunogenic tumor cell stress or death and coordinate innate and adaptive immune activation within the TME. Physical, chemical, and biologic modalities can fulfill this goal by promoting antigen release or availability in the context of inflammatory signaling that supports antigen presentation and T-cell priming. Representative clinical trials and quantitative outcomes are summarized in **Table 1**, whereas the major ISV strategies, their primary roles, evidence levels, and key limitations are summarized in **Table 2** and **Figure 1**.

**Table 1:** Representative clinical trials of *in situ* vaccination strategies.

ISV modality	Indication	ISV trigger/combination	Trial phase	Primary/key endpoints	Key clinical & immunologic findings	Ref
Low-dose RT + intratumoral CpG	Low-grade B-cell lymphoma	RT 2 Gy × 2 to one lesion + IT PF-3512676–6 mg (before RT, after the second RT fraction, then weekly × 8; 10 injections total)	Phase I/II n = 15	Safety; response at distant untreated sites; tumor-reactive T-cell response	Distant lesions: 1 CR + 3 PRs (ORR 26.7%); 2 additional prolonged, continuously regressing SDs. Tumor-reactive memory CD8+ T cells increased. AEs were grade 1–2; no treatment-limiting toxicity (small single-arm cohort).	(35)
Low-dose RT + intratumoral TLR9 agonist	Untreated indolent lymphoma	RT 2 Gy/day on days 1–2 (4 Gy total) + five weekly IT SD-101 injections (1, 2, 4, or 8 mg)	Phase I/II n = 29	Safety; treated- and untreated-site response; immune correlates	Untreated-site burden decreased in 24/29 (82.8%); 1 CR + 5 PRs (systemic ORR 20.7%); nearly all treated lesions regressed. No treatment-related grade 4 or serious AEs (single-arm).	(82)
Flt3L–RT–poly-ICLC	Advanced indolent non-Hodgkin lymphoma	IT Flt3L 25 μg/kg daily × 9 → low-dose RT 2 Gy × 2 → IT poly-ICLC 2 mg × 8	Phase I/II; initial cohort n = 11	Local and systemic tumor response; tumor-reactive T-cell priming; resistance mechanisms	Eight of 11 treated lesions had partial or complete regression; untreated lesions showed 1 CR, 2 PRs, 6 SD/minor regressions and 2 PD (systemic ORR 27.3%). One grade 2 fever occurred; all other AEs were grade 1 (small mechanistic cohort).	(75)
RT + PD-1 blockade (PEMBRO-RT)	Advanced NSCLC	SBRT 8 Gy × 3 to one lesion, followed by pembrolizumab vs pembrolizumab alone	Randomized phase II n = 76	ORR at 12 weeks; PFS; OS; safety	ORR was 36% vs 18% (P = 0.07); median PFS 6.6 vs 1.9 months and OS 15.9 vs 7.6 months, neither significant. Grade 3–5 toxicity was not increased.	(38)
RT + intratumoral TLR4 agonist (GLA-SE)	Metastatic soft-tissue sarcoma	IT GLA-SE 5 or 10 μg weekly × 8 + concurrent RT to the injected lesion	Phase I, nonrandomized n = 12	Safety; injected-lesion response; systemic TCR expansion	All 12 injected lesions were controlled (1 CR; mean change −25%, range −100% to +4%). Matched intratumoral and circulating TCR-clone expansion was detected in selected patients, but no systemic RECIST responses occurred. One grade 2 TRAE; no grade ≥3 events.	(83)
Cryoablation ± ipilimumab	Early-stage breast cancer before mastectomy	Preoperative cryoablation alone (n = 7), single-dose ipilimumab 10 mg/kg alone (n = 6), or the combination (n = 6)	Pilot study n = 19	Feasibility; safety; systemic and intratumoral immune activation	No surgery was delayed. Combination therapy induced sustained Th1 cytokines, activated/proliferating CD4+ and CD8+ T cells, and a higher intratumoral proliferating effector:Treg ratio. One unrelated grade 3 rash occurred (mechanistic pilot; not powered for efficacy).	(41)
T-VEC monotherapy	Unresectable stage IIIB–IV melanoma	IT T-VEC vs subcutaneous GM-CSF	Randomized phase III n = 436	DRR; ORR; OS; safety; injected and uninjected lesion response	DRR 16.3% vs 2.1%; ORR 26.4% vs 5.7%; median OS 23.3 vs 18.9 months (HR 0.79; P = 0.051). Complete resolution: 47% injected, 22% uninjected nonvisceral, 9% visceral lesions. Cellulitis was the only grade 3–4 AE ≥2% (2.1%).	(53, 94)
T-VEC + ipilimumab	Advanced unresectable melanoma	IT T-VEC every 2 weeks + ipilimumab 3 mg/kg every 3 weeks (≤4 doses) vs ipilimumab alone	Randomized phase II n = 198	ORR; uninjected/visceral-lesion response; safety	ORR 39% (38/98) vs 18% (18/100; P = 0.002); visceral-lesion decreases 52% vs 23%. Grade ≥3 AEs occurred in 45% vs 35% (open-label).	(55)
T-VEC + pembrolizumab (phase Ib)	Advanced melanoma	IT T-VEC followed by combination with pembrolizumab	Phase Ib n = 21	ORR; CR; safety; tumor immune remodeling	Confirmed ORR 61.9%; CR 33%; no dose-limiting toxicity. Responses correlated with increased intratumoral CD8+ T cells and higher PD-L1 and IFN-γ gene expression (small, noncomparative phase Ib).	(54)
T-VEC + pembrolizumab (confirmatory trial)	Advanced melanoma	IT T-VEC + pembrolizumab vs placebo + pembrolizumab	Randomized phase III n = 692	PFS; OS; ORR; DRR; safety	No significant PFS (HR 0.86; P = 0.13) or OS benefit (HR 0.96; P = 0.74). ORR 48.6% vs 41.3%; DRR 42.2% vs 34.1%. Grade ≥3 TRAEs were similar (20.7% vs 19.5%).	(84)
Neoadjuvant T-VEC	Resectable stage IIIB–IVM1a melanoma	Six neoadjuvant IT T-VEC doses followed by surgery vs immediate surgery	Randomized phase II n = 150	RFS; OS; pCR; safety	Two-year RFS 29.5% vs 16.5% (HR 0.75); 2-year OS 88.9% vs 77.4% (HR 0.49); pCR 17.1% (open-label phase II, surgery-based setting).	(56)
Intratumoral SD-101 + pembrolizumab	Unresectable or metastatic melanoma	IT SD-101 + pembrolizumab	Phase Ib n = 22	Safety; ORR; PFS; immune activation	Anti-PD-1-naive: ORR 78% (7/9), estimated 12-month PFS 88%; previously treated: ORR 15% (2/13). AEs were mainly injection-site reactions and transient mild-to-moderate flu-like symptoms (small nonrandomized cohort).	(85)
Intratumoral STING agonist (MIW815/ADU-S100)	Advanced/metastatic solid tumors or lymphoma	IT MIW815 50–6,400 μg weekly (3 weeks on/1 week off)	Phase I n = 47	Safety; pharmacodynamics; injected-lesion response	One confirmed and two unconfirmed PRs; 94% of evaluable injected lesions were stable or smaller. Common TRAEs: pyrexia 17%, chills 15%, injection-site pain 15%. Pharmacodynamic activation occurred, but single-agent activity was limited.	(58)
Intratumoral IL-12 gene delivery (TAVO-EP + nivolumab)	Operable locoregionally advanced melanoma	IT tavokinogene telseplasmid electroporation + IV nivolumab (up to three 4-week neoadjuvant cycles), followed by surgery	Phase II, single arm n = 16	Radiologic response; MPR; pCR; recurrence	Radiologic response 63%. Among 15 resected patients, MPR 80% (12/15) and pCR 60% (9/15); at a median follow-up of 15.4 months, no patient with MPR had recurred (small single-arm neoadjuvant study).	(64)
OX40 agonist added to *in situ* vaccination	Low-grade B-cell lymphoma	Low-dose RT 2 Gy × 2 + IT SD-101 × 5 + IT/IV OX40 agonist BMS-986178	Phase I n = 14	Safety; ORR; PFS; immune activation	Best responses: 1 PR, 9 SD, 4 PD; no dose-limiting or synergistic toxicity. Activity was lower than in earlier RT + SD-101 studies, indicating limited clinical translation of preclinical OX40 synergy (small phase I).	(66)
Intratumoral CD40 agonist (ADC-1013)	Advanced solid malignancies	ADC-1013 IT 22.5–400 μg/kg or IV 75 μg/kg every other week	Phase I n = 23	Safety; pharmacodynamics; dose escalation	AEs were mainly transient grade 1–2. Peripheral B cells markedly decreased and CD86 increased on remaining B cells, confirming pharmacodynamic activity; objective antitumor efficacy was not established (small dose-escalation cohort; mixed IT/IV dosing).	(65)
Intratumoral BO-112 + pembrolizumab	Anti-PD-1-resistant advanced melanoma	IT BO-112 (up to 2 mg in up to 8 lesions) weekly × 7, then every 3 weeks + pembrolizumab 200 mg every 3 weeks	Phase II, single arm n = 42	ORR; DOR; PFS; OS; safety	ORR 25% (CR 10%, PR 15%); SD 40%; median PFS 3.7 months; 54% were alive at 24 months. Grade 3–4 AEs occurred in 38.1% overall and were drug-related in 9.5%; no treatment-related deaths (single-arm).	(96)

**Table 2:** Major *in situ* vaccination strategies and platforms.

ISV strategy	Representative modalities, agents, or platforms	Primary role in ISV	Evidence level and key limitations	Ref
Radiotherapy	Focal RT/SBRT; low-dose RT plus CpG/SD-101; RT plus ICB	Promotes antigen release and cGAS–STING–IFN-I signaling.	Preclinical to randomized clinical evidence; dose and fractionation dependence and inconsistent systemic benefit.	(33, 34, 38, 82)
Tumor ablation and other energy-based modalities	Cryoablation; RFA; HIFU; PTT/PDT	Releases tumor material through freeze–thaw, thermal, mechanical or ROS-mediated injury.	Strong preclinical and limited early clinical evidence; variable adjuvanticity and technique-dependent immune effects.	(13, 41–44)
ICD-inducing chemotherapy	Anthracyclines; oxaliplatin; metronomic cyclophosphamide; local delivery	Selected agents expose CRT and release ATP/HMGB1, supporting DC activation.	Strong mechanistic and preclinical evidence; limited stand-alone ISV evidence, poor spatial specificity and systemic toxicity.	(6, 45–47)
Oncolytic viruses	T-VEC; oncolytic measles or Newcastle disease viruses; transgene-encoding vectors	Combines tumor lysis, antigen release and virus-driven innate immune activation.	Established clinical evidence for T-VEC; antiviral immunity, limited intratumoral spread and mixed combination results.	(49, 51, 53, 55, 56)
Intratumoral innate immune agonists	CpG/SD-101; poly-ICLC; GLA-SE/G100; STING agonists	Activates local innate sensing, DC maturation and T-cell cross-priming.	Preclinical to phase I/II evidence; TLR agonists are more clinically developed than STING agonists, and activity is formulation- and context-dependent.	(17, 35, 58, 75, 82, 85)
Cytokines and costimulatory agonists	IL-12 electroporation; local cytokine delivery; OX40, CD40 or 4-1BB agonists	Enhances APC function and supports T-, NK- or myeloid-cell activation.	Preclinical to early clinical evidence; systemic leakage or toxicity and sequence- and receptor-dependent activity.	(62–66, 86)
Nucleic-acid platforms	Intratumoral mRNA/mRNA-LNP combinations; nanoplexed poly-IC (BO-112)	Provides transient local expression of immune mediators or activates dsRNA sensing.	Predominantly preclinical; BO-112 has single-arm phase II evidence. Delivery, stability and manufacturing remain barriers.	(69, 70, 78, 96)
Biomaterial-enabled delivery platforms	LNPs; injectable hydrogels or cryogels; biodegradable depots	Improves local retention, controlled release and co-delivery of ISV agents.	Predominantly preclinical; scale-up, batch reproducibility and device–drug regulatory requirements limit translation.	(61, 67, 68)
Emerging mitochondria-targeted agents	Mitochondria-targeted atovaquone (Mito-ATO)	Reprograms immune-cell metabolism and reduces suppressive immune populations.	Preclinical only; evidence is platform-specific and human safety and efficacy remain untested.	(73, 74)
Multimodal combinations	Flt3L → low-dose RT → poly-ICLC; RT or ablation plus immune agonists/ICB	Combines antigen release, APC recruitment or activation, and checkpoint relief.	Strong preclinical rationale and selected clinical evidence; dose and sequence complexity and mixed randomized results.	(38, 44, 75, 82, 84)

**Figure 1:**
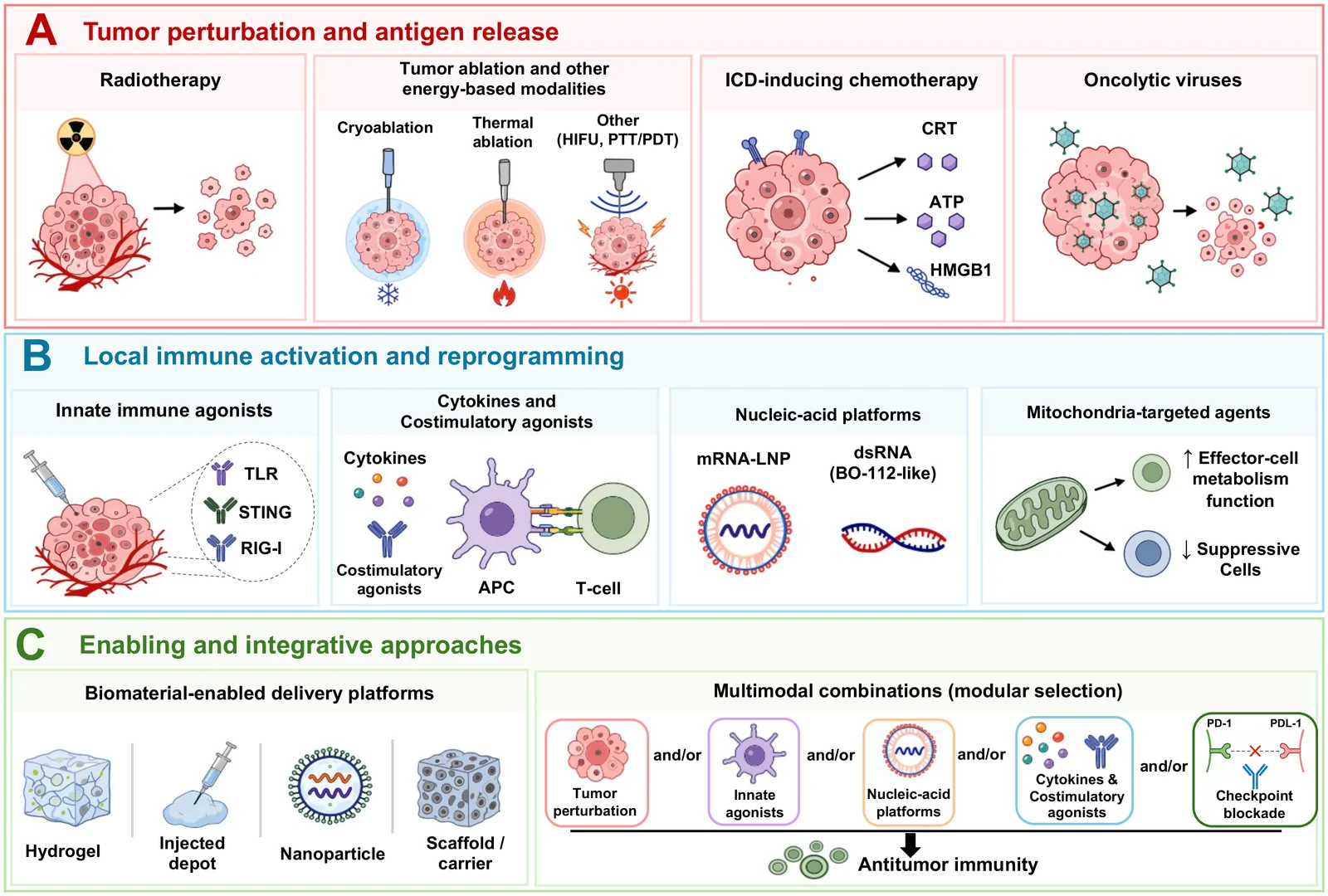
*In situ* vaccination strategies and platforms. **(A)** Radiotherapy, tumor ablation and other energy-based modalities, selected ICD-inducing chemotherapies, and oncolytic viruses increase tumor-antigen release through tumor-cell stress or death. **(B)** Intratumoral innate immune agonists, cytokines and costimulatory agonists, nucleic-acid platforms, and emerging mitochondria-targeted agents activate or reprogram immune cells within the tumor microenvironment. The rightmost schematic illustrates metabolic reprogramming by mitochondria-targeted agents. Nucleic-acid approaches include mRNA–LNP formulations for local protein expression and double-stranded RNA platforms such as BO-112. **(C)** Biomaterial-enabled delivery platforms improve intratumoral retention and controlled release, whereas multimodal treatments bring together complementary mechanisms of antigen release, immune activation, and sustained T-cell activity.

### Radiation-based *in situ* vaccination

RT is the most extensively studied ISV modality. Beyond direct cytotoxicity, RT induces DNA damage and cellular stress responses that promote ICD, antigen release, and innate immune sensing. RT-induced tumor-cell stress and death can also expose calreticulin (CRT) and release extracellular ATP and high-mobility group box 1 (HMGB1), thereby promoting the recruitment of APCs and phagocytic uptake of tumor-derived material by dendritic cells (6, 27). Ionizing radiation promotes the accumulation of cytosolic double-stranded DNA, which activates the cyclic GMP–AMP synthase (cGAS)–stimulator of interferon genes (STING) pathway in tumor cells and APCs (33). The resulting IFN-I signaling supports DC maturation, cross-priming, and effective CD8^+^ T-cell responses. RT also upregulates MHC class I and adhesion molecules on tumor cells, thereby enhancing immune recognition. These immunological effects are highly dependent on dose and fractionation: fractionated regimens can sustain IFN-I signaling and immune priming, whereas very high single-fraction doses induce the exonuclease TREX1, which degrades cytosolic DNA and attenuates cGAS–STING activation (33, 34). Consistent with this mechanism, fractionated RT at 8 Gy × 3 combined with anti-CTLA-4 induced abscopal responses in a bilateral mammary carcinoma model, whereas single high-dose RT was ineffective (34). These findings underscore that local tumor control does not necessarily translate into systemic antitumor immunity and provide a mechanistic rationale for optimizing RT fractionation in combination with ICB.

Preclinical studies further demonstrate that host STING-dependent IFN-I signaling is required for RT-induced antitumor immunity and that combining RT with checkpoint blockade can enhance control of distant tumors (10, 11, 33). Clinically, RT-based ISV has been evaluated in combination with intratumoral TLR agonists and ICB; detailed trial findings are discussed in the Clinical ISV Studies section (35–38).

### Tumor ablation and energy-based modalities

Local tumor ablation techniques including cryoablation, radiofrequency ablation, microwave ablation, high-intensity focused ultrasound (HIFU), photothermal therapy (PTT), and photodynamic therapy (PDT) represent another major class of ISV strategies (13, 39–42). These methods destroy tumor cells through thermal, mechanical, or oxidative stress, resulting in tumor-antigen release. The resulting tumor-derived material can be taken up by dendritic cells through phagocytosis, supporting antigen processing and cross-presentation to tumor-reactive T cells (43). Cryoablation can preserve cellular architecture and antigen integrity while inducing necrotic cell death that becomes highly immunogenic in the presence of adequate inflammatory cues (39–41). By contrast, heat-based ablation may denature proteins and reduce antigen quality if not carefully optimized. PDT and some PTT platforms combine localized cytotoxicity with reactive oxygen species (ROS) generation, promoting endoplasmic reticulum stress, DAMP exposure, and innate immune activation. However, because ablation alone often provides insufficient adjuvanticity, combination with intratumoral immune agonists or systemic ICB may improve efficacy. Preclinical studies suggest that such combinations can enhance systemic antitumor immunity, although clinical evaluation remains at an early stage (41, 44).

### Chemotherapy-induced *in situ* vaccination

Although chemotherapy is traditionally regarded as immunosuppressive, selected agents can induce ICD and function as an enabling component of ISV. Anthracyclines, oxaliplatin, and cyclophosphamide are among the best-characterized examples (14, 45). Anthracyclines and oxaliplatin can promote CRT exposure, ATP secretion, and HMGB1 release. Surface-exposed CRT facilitates phagocytic uptake of dying tumor cells by DCs, while extracellular ATP and HMGB1 activate P2RX7–NLRP3 and TLR4-dependent pathways, respectively, supporting DC activation, antigen processing, and cross-presentation (27, 46, 47). Their immunogenicity is strongly context-dependent and influenced by dose and schedule. Preclinical studies demonstrate that disruption of CRT exposure or host DAMP-sensing pathways impairs chemotherapy-induced antitumor immunity (46, 47). Metronomic cyclophosphamide may reduce regulatory T-cell-mediated suppression, whereas localized or biomaterial-enabled delivery may increase intratumoral exposure and limit systemic toxicity (48). However, systemic chemotherapy lacks spatial specificity and may impair immune effector populations. Clinically, chemotherapy is generally incorporated into multimodal regimens, and evidence supporting chemotherapy as a stand-alone ISV remains limited (45).

### Oncolytic viruses as biologic ISV platforms

Oncolytic viruses (OVs) are a clinically advanced biologic ISV modality. These engineered or naturally occurring viruses selectively infect and lyse tumor cells, releasing tumor antigens while activating innate antiviral immunity (49, 50). Viral replication generates pathogen-associated molecular patterns (PAMPs) that stimulate PRRs, including TLRs and cytosolic RNA and DNA sensors, resulting in robust IFN-I responses. Following oncolysis, tumor-cell debris containing tumor-derived antigens can be taken up by DCs through phagocytosis, enabling antigen processing and cross-presentation to tumor-specific CD8^+^ T cells in the context of virus-induced inflammatory signaling (51). This intrinsic adjuvanticity distinguishes OVs from other ISV approaches. In addition, OVs can be engineered to express immunostimulatory transgenes such as GM-CSF, IL-12, or costimulatory ligands to further enhance antigen presentation and T-cell priming. In preclinical models, Flt3L-mediated expansion of cross-presenting DCs enhanced oncolytic Newcastle disease virus–induced tumor-antigen uptake, CD8^+^ T-cell cross-priming, and durable tumor control (52). Oncolytic viro-immunotherapy represents the most mature clinical application of ISV principles: talimogene laherparepvec (T-VEC) improved durable response rates in melanoma and induced regression in uninjected lesions, while early combination studies with ICB showed encouraging activity (53–55). Neoadjuvant T-VEC has also been linked to improved recurrence-free survival (56). Nonetheless, antiviral immunity, pre-existing neutralizing antibodies, and limited intratumoral spread remain important barriers to efficacy and durability.

### Intratumoral delivery of immune agonists

Direct intratumoral administration of innate immune agonists is a versatile ISV strategy. Agonists targeting TLRs, STING, RIG-I–like receptors, and related PRRs can directly activate DCs and macrophages within the TME (57). This activation induces IFN-I and pro-inflammatory cytokines and enhances co-stimulatory and antigen-presentation programs in antigen-bearing DCs, thereby supporting the cross-priming of tumor-reactive T cells (17, 57). TLR9 agonists (CpG oligodeoxynucleotides), TLR3 agonists such as poly(I:C), and STING agonists have shown strong preclinical efficacy, especially when combined with local tumor disruption or systemic ICB. In preclinical models, intratumoral STING agonism induced regression of treated tumors, rejection of distant metastases, and long-lived immunologic memory (17). Because these agents do not depend on tumor cell infection or replication, they offer more predictable pharmacology and rapid immune activation. Intratumoral delivery also permits high local concentrations while limiting systemic toxicity, which has constrained clinical development. However, dose optimization remains essential because excessive innate activation may cause local tissue damage or systemic inflammatory toxicity (57, 58). Clinically, intratumoral TLR9 agonists have induced systemic responses in lymphoma, whereas early STING agonist trials have demonstrated pharmacodynamic immune activation but limited single-agent antitumor activity (35, 58).

Cytokines and costimulatory ligands can also be delivered intratumorally to shape the magnitude and quality of ISV-induced immunity. GM-CSF supports DC recruitment and differentiation, IL-2 and IL-15 promote T-cell expansion and persistence, and IL-12 drives Th1 polarization and cytotoxic effector function (59, 60). Although systemic administration is often limited by toxicity, local delivery can enable strong immune modulation with better tolerability (18, 61). Likewise, intratumoral agonistic antibodies targeting OX40, CD137 (4-1BB), or CD40 can enhance T-cell priming and overcome local immune suppression. Intratumoral CpG combined with OX40 agonism has demonstrated antitumor activity in lymphoma, breast, and colon cancer models, with enhanced efficacy in neoadjuvant and optimized perioperative settings (5, 62, 63). The efficacy of these combinations depends strongly on treatment timing and sequencing, particularly relative to antigen release and APC activation. Early-phase clinical studies suggest that intratumoral cytokines and costimulatory agonists are feasible and can induce immune activation, although their clinical efficacy remains to be established and may require combination with other therapies (64–66).

### Nucleic acid–based and biomaterial-enabled ISV platforms

Recent advances in delivery science have expanded the ISV toolkit to include nucleic acids and biomaterial scaffolds (67, 68). Emerging intratumoral mRNA and LNP–mRNA platforms have shown the potential to enhance responses to PD-1 blockade in preclinical models (69, 70). Intratumoral mRNA encoding cytokines, costimulatory ligands, or tumor antigens enables transient, localized protein expression without genomic integration (**Figure 1B**) (69–71). LNP encapsulation protects mRNA from degradation and facilitates cellular uptake and protein expression within the TME. In preclinical tumor models, intratumoral mRNA combinations encoding IL-23, IL-36γ, and OX40L or IL-21, IL-7, and 4-1BBL induced tumor regression and durable antitumor immunity (69, 70). Advances in delivery technologies including lipid nanoparticles, injectable hydrogels, and biodegradable scaffolds now enable spatial and temporal control of immune activation within the TME while minimizing systemic exposure (67, 68, 71). These innovations align with the ‘HIT-IT (human intratumoral immunotherapy)’ paradigm. These systems can co-deliver antigens and adjuvants, effectively recreating vaccine architecture *in situ* (67, 68, 72). Their modularity makes them especially attractive for combination ISV strategies across tumor types.

### Mitochondria-targeted agents

Mitochondria-targeted agents can enhance immunogenicity by altering oxidative metabolism or promoting ROS accumulation to induce apoptosis and metabolic immune cell reprogramming. The mitochondria-targeted atovaquone derivative Mito-ATO exerts antitumor activity primarily through reprogramming of the TME rather than through direct tumor-cell killing. In preclinical models, Mito-ATO acts as an ISV by triggering systemic T-cell immunity and improving the antitumor effect of PD-1 blockade (73). Mechanistically, Mito-ATO exerts opposing metabolic effects on antitumor and immunosuppressive immune-cell populations. In cytotoxic CD4^+^ T cells, effector CD8^+^ T cells, and M1 macrophages, Mito-ATO enhances OXPHOS, the TCA cycle, and pyruvate metabolism, whereas in G-MDSCs and Tregs it suppresses these pathways, impairing their survival (74). These opposing metabolic effects reduce immunosuppression and enhance antitumor immunity, supporting the use of Mito-ATO in combination with ICB (**Figure 1B**).

### Combination and multimodal ISV strategies

Accumulating evidence indicates that no single ISV modality is universally sufficient for durable systemic immunity. The most effective regimens therefore combine complementary elements: trigger of ICD, a source of innate immune activation, and relief of adaptive immune suppression through ICB. Multimodal strategies, such as RT with intratumoral TLR or STING agonists plus systemic PD-1 blockade, have shown synergistic efficacy in preclinical models and early clinical trials (17, 33, 75, 76). These combinations increase antigen availability, enhance DC activation and T-cell priming, and sustain effector T-cell activity at both local and distant sites. Rational design requires careful attention to dose, sequence, tumor accessibility, and immune contexture. As mechanistic understanding and delivery technologies continue to advance, ISV is increasingly viewed as a combination platform rather than a single therapeutic intervention (**Figure 1C**).

### Comparison with traditional cancer vaccines

ISV differs fundamentally from conventional cancer vaccines. Traditional vaccines require prior identification, isolation, and formulation of selected tumor antigens with defined adjuvants before administration. Although this allows precise antigen targeting, it is constrained by tumor heterogeneity, antigen loss, immune escape, and tolerance to self-derived antigens (77). In contrast, ISV exploits ICD to expose the full antigenic landscape of the tumor directly within the host. By eliminating the need for antigen selection and centralized vaccine manufacturing, ISV reduces logistical complexity while enabling a broader and more personalized immune response (20). These features make ISV particularly well suited for malignancies without well-defined target antigens.

## Preclinical evidence and mechanistic insights

Preclinical studies across diverse tumor models have been instrumental in defining the immunologic principles underlying ISV (62, 63, 68). Modality-specific mechanisms and preclinical evidence are described in the preceding sections, whereas this section summarizes mechanistic principles shared across ISV strategies. Collectively, these studies demonstrate that durable systemic antitumor immunity arises not from tumor destruction alone, but from the coordinated induction of ICD or other forms of immunogenic tumor cell stress or death, activation of innate immune sensing pathways, and effective DC–mediated T-cell priming. These mechanistic elements (**Figure 2**) form the foundation upon which modality-specific ISV strategies are built (**Figure 1**).

**Figure 2:**
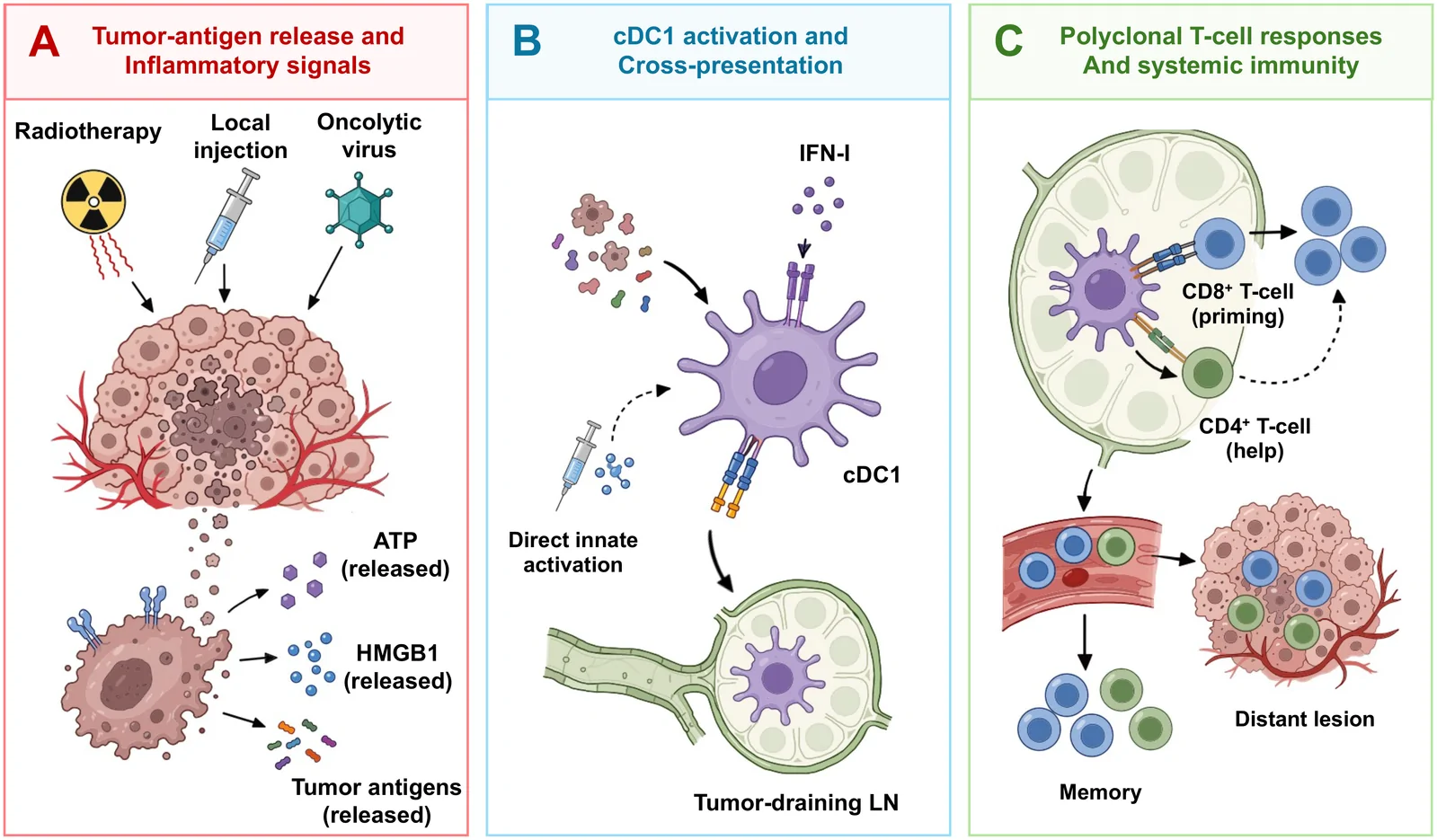
Immunological mechanism of *in situ* vaccination. **(A)** Local treatment releases or exposes tumor antigens together with inflammatory signals. In treatments that induce immunogenic cell death, calreticulin is exposed on dying tumor cells, whereas ATP and HMGB1 are released into the tumor microenvironment. **(B)** cDC1s take up tumor-derived antigens and migrate to the tumor-draining lymph node, where they cross-present these antigens following IFN-I signaling. **(C)** Activated tumor-reactive CD8^+^ T cells, supported by CD4^+^ T cells, enter the circulation and reach both treated and untreated lesions. Epitope spreading and memory formation can broaden and prolong the antitumor response.

### Induction of immunogenic cell death and antigen availability

Multiple ISV triggers including RT, OVs, ablation, PDT, and selected chemotherapeutics have been shown to induce ICD in preclinical cancer models (6, 13, 39, 45, 49, 50). Hallmarks of ICD, including surface CRT exposure, ATP release and HMGB1 secretion, consistently correlate with enhanced antigen uptake by DCs and improved T-cell priming. Genetic or pharmacological disruption of these pathways can impair antitumor immunity, confirming their causal role in ISV efficacy (46, 47). Notably, preclinical models demonstrate that the quality of antigen release is as important as quantity. Modality-specific treatment parameters can influence the immunogenic context of tumor-antigen release. For example, RT dose and fractionation regulate cytosolic DNA sensing and IFN-I signaling, whereas cryoablation and radiofrequency ablation differ in the extent of DC antigen loading (33, 34, 43). These findings provide mechanistic rationale for modality selection and parameter optimization (**Figure 1A**).

### cGAS–STING and type I interferon signaling as central hubs

A recurring theme in preclinical ISV studies is the central role of the cGAS–STING–IFN-I axis in linking tumor cell death to adaptive immunity (**Figure 2B**). Tumor-derived cytosolic DNA generated by RT, viral infection, or regulated necrosis activates STING signaling in both tumor cells and host APCs, driving IFN-I production. In preclinical RT models, loss of host STING signaling attenuates IFN-I production and antitumor immunity (33). Genetic ablation of STING or IFNAR1 in host immune cells markedly impairs DC maturation, cross-presentation, and CD8^+^ T-cell priming, resulting in loss of abscopal tumor control (17, 33). Conversely, intratumoral STING agonists can induce local and systemic antitumor responses in preclinical models (17). These findings position STING signaling as a non-redundant mechanistic node and rational target for ISV enhancement (**Figure 1B**).

### Dendritic cell subsets and cross-priming requirements

Elegant genetic and depletion studies have established cDC1s as essential mediators of ISV-induced T-cell immunity (7–9, 79). In multiple tumor models, cDC1 deficiency abolishes CD8^+^ T-cell priming and eliminates distant tumor regression despite intact local tumor destruction (7, 9, 79). Preclinical imaging and lineage-tracing experiments further reveal that cDC1s acquire intact tumor antigens *in situ*, migrate to tumor-draining lymph nodes, and initiate cross-priming under IFN-I–rich conditions (**Figure 2B**) (9, 33). Strategies that expand or activate cross-presenting DCs, such as Flt3L-based ISV regimens or CD40 agonism, have been shown to enhance T-cell priming and systemic antitumor responses (75, 80).

### Epitope spreading and polyclonal T-cell responses

A defining feature of effective ISV in preclinical models is the induction of epitope spreading, whereby initial immune responses against dominant tumor antigens evolve into broader polyclonal T-cell responses to subdominant antigenic targets on tumor cells (81). This phenomenon is linked to sustained antigen release, efficient DC cross-presentation, and iterative rounds of T-cell priming. Functionally, epitope spreading may confer resilience against antigen-loss variants and tumor immune escape (81). Rechallenge experiments have shown that animals cured by ISV reject subsequent tumor challenge and that epitope spreading can extend protection to tumors lacking the initiating neoantigens, underscoring the breadth and durability of ISV-induced immunity (**Figure 2C**) (5, 17, 63, 81).

### Abscopal effects and systemic immune surveillance

Preclinical models provide compelling mechanistic evidence for the abscopal effect as an immune-mediated phenomenon rather than a passive consequence of tumor debulking (10, 11). In bilateral tumor models, localized ISV treatment induces CD8^+^ T-cell–dependent regression of untreated lesions, accompanied by increased T-cell infiltration at distant tumor sites and enhanced tumor-specific IFN-γ responses (11, 17, 62). Importantly, abscopal responses are markedly attenuated in immunodeficient hosts or in the absence of adaptive immunity and can be potentiated by ICB (10–12).

### Immune memory and long-term protection

Beyond acute tumor regression, preclinical ISV studies consistently demonstrate the establishment of long-term immune memory. Animals cured of primary tumors resist rechallenge more than 100 days after tumor regression in CpG-based models and 60 days after initial tumor inoculation in models treated with intratumoral STING agonists (5, 17). Memory formation has been associated with the persistence of tumor-reactive central and effector memory T cells and, in some preclinical models, with tumor-specific antibody responses. The durability of immune protection distinguishes ISV from purely cytotoxic local therapies and provides a mechanistic basis for its evaluation in curative intent and neoadjuvant settings (**Figure 2C**) (5, 17).

### Context dependence and resistance mechanisms

Preclinical studies also highlight key resistance mechanisms that constrain ISV efficacy. These include insufficient DC recruitment, defective IFN-I signaling, enrichment of immunosuppressive myeloid populations, regulatory T cells and exclusion of effector T cells from the TME. Importantly, these barriers are not immutable. Combination strategies incorporating myeloid modulation, cytokine delivery, or checkpoint inhibition can reprogram resistant tumors and enhance ISV responsiveness, indicating that ISV efficacy is shaped by immune contexture rather than tumor cell–intrinsic sensitivity alone (**Figure 3**) (11, 17, 33, 75).

**Figure 3:**
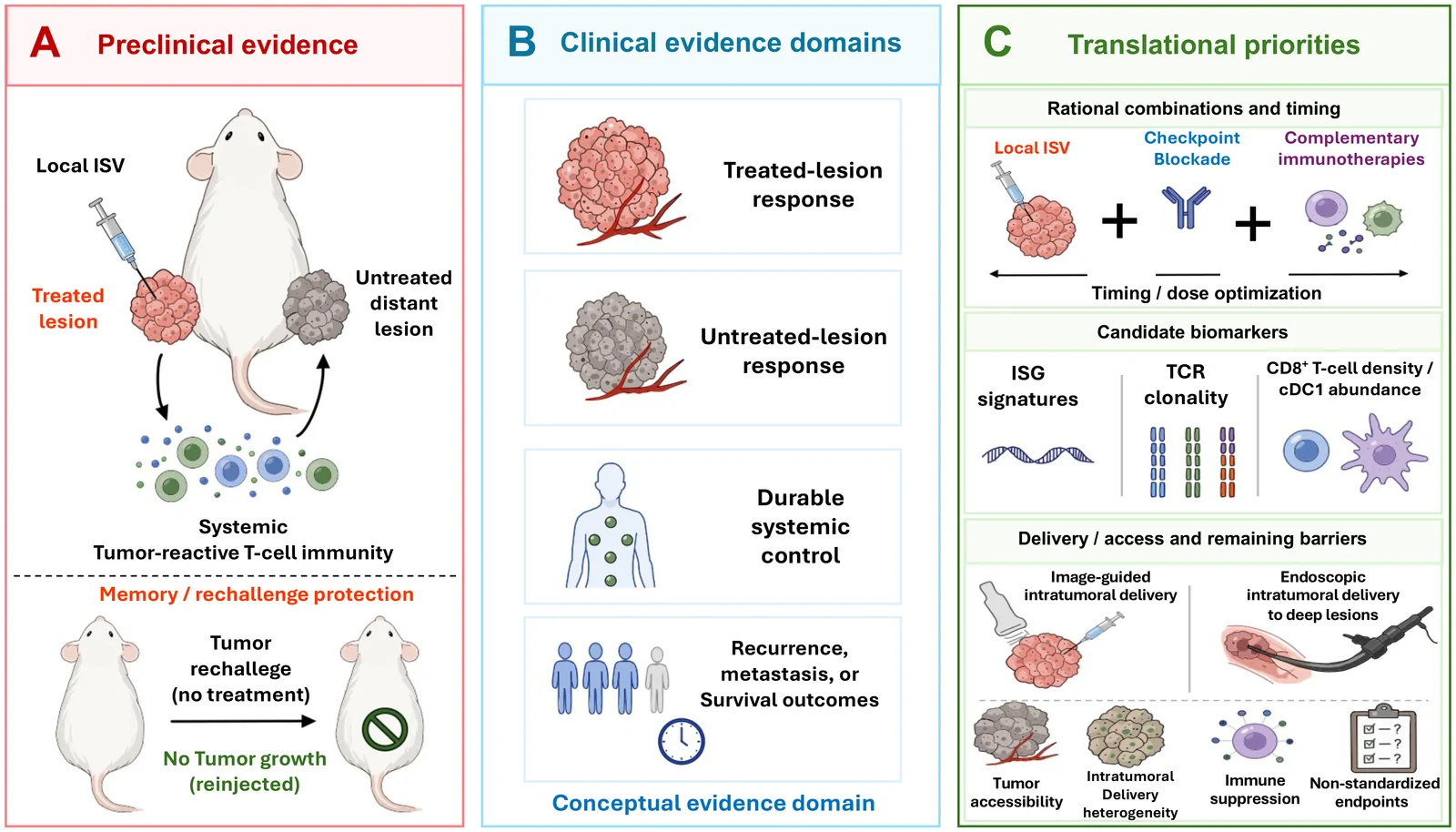
Preclinical-to-clinical translation of *in situ* vaccination. **(A)** Preclinical models with a treated tumor and an untreated distant lesion are used to determine whether local ISV can generate systemic T-cell responses and control untreated tumors. Tumor rechallenge experiments are used to assess immune memory. **(B)** Clinical evidence includes responses in treated and untreated lesions, durable systemic disease control, and effects on recurrence, metastasis, and survival. These outcomes are related but measure different aspects of treatment benefit. **(C)** Clinical development requires optimization of treatment combinations, dose and sequencing, biomarker monitoring, and delivery to deep or difficult-to-access tumors. Tumor accessibility, uneven intratumoral delivery, immune suppression, and the lack of standardized endpoints remain important challenges.

## Clinical ISV studies

Early clinical studies support the translational potential of ISV, with evidence that localized immune priming can generate systemic antitumor responses across selected tumor types, particularly when combined with ICB (35, 53, 82, 83). Clinical studies are stratified by ISV modality, with emphasis on trial endpoints, biomarkers, and translational insights. Representative clinical trials across key ISV modalities, including quantitative efficacy, immunologic and safety outcomes and key study limitations, are summarized in **Table 1** and **Figure 3**.

### Radiotherapy

RT has served as the most widely tested clinical ISV trigger, owing to its ubiquity, predictable local control, and immunomodulatory potential. Early phase studies combining focal RT with intratumoral or systemic immune adjuvants established feasibility and provided clinical evidence of abscopal immune effects (12). In indolent non-Hodgkin lymphoma and mycosis fungoides, low-dose RT combined with intratumoral CpG induced systemic immune responses and regression of nonirradiated lesions with manageable toxicity (35, 36). Primary endpoints focused on safety and local response, while secondary endpoints included distant tumor responses and immune correlates, supporting the ISV premise in humans. Subsequent studies demonstrated immune activation, including increased tumor-specific T cells and cytokine signatures consistent with IFN-I engagement (35, 36, 82). In solid tumors, RT has most commonly been paired with ICB. Trials in melanoma, non–small cell lung cancer, and head and neck squamous cell carcinoma have reported responses in irradiated and non-irradiated lesions, with higher response rates than those reported in historical controls. While objective abscopal responses remain infrequent, exploratory endpoints, including T-cell receptor (TCR) clonality expansion and intratumoral CD8^+^ T-cell infiltration, support immune-mediated systemic effects (37, 38). These findings underscore the importance of endpoint selection that extends beyond RECIST based response criteria, particularly in early-phase ISV trials.

### Oncolytic virus

Oncolytic virus immunotherapy represents the most mature clinical application of ISV principles. T-VEC, an HSV-1–based OV encoding GM-CSF, was the first oncolytic virus therapy to receive regulatory approval. In a randomized phase III trial in advanced melanoma, T-VEC improved the durable response rate (DRR) compared with GM-CSF alone (53). Notably, responses were observed in both injected and noninjected lesions, providing clinical evidence of systemic antitumor activity. Secondary endpoints included overall response rate (ORR) and safety, with immune-related adverse events comparable to other immunotherapies. Early combination trials pairing T-VEC with ICB further strengthened the ISV paradigm. In a randomized phase II study, T-VEC plus ipilimumab improved ORR relative to ipilimumab alone, whereas T-VEC plus pembrolizumab was associated with objective and complete responses in a phase Ib cohort without a monotherapy comparator (54, 55). However, a subsequent randomized phase III trial of T-VEC plus pembrolizumab did not significantly improve progression-free or overall survival compared with placebo plus pembrolizumab (84). These apparently discordant results should be interpreted in the context of important differences among the studies. The randomized phase II study evaluated T-VEC plus ipilimumab and was therefore not directly comparable with the pembrolizumab phase III trial (55, 84). In the phase Ib pembrolizumab study, pembrolizumab was initiated at week 6 after two preceding doses of T-VEC, whereas T-VEC/placebo and pembrolizumab were initiated simultaneously in the phase III study (54, 84). In the phase Ib study, translational endpoints revealed increased CD8^+^ T-cell infiltration and increased expression of interferon-stimulated genes in tumors following T-VEC treatment. These data support a model in which local viral oncolysis primes antitumor immunity, while ICB sustains T-cell effector function. These differences highlight the potential importance of treatment sequence and clinical context in OV–ICB combinations and warrant further prospective evaluation. Neoadjuvant application of T-VEC has extended ISV into earlier disease settings. In a randomized phase II trial in resectable melanoma, neoadjuvant T-VEC improved recurrence-free survival compared with surgery alone, supporting the potential of ISV as a preoperative immune-priming strategy (56).

### Intratumoral innate immune agonists

Clinical trials of intratumoral innate immune agonists have included administration of TLRs and STING agonists (35, 58). CpG oligodeoxynucleotides (TLR9 agonist) have been the most extensively studied, particularly in lymphoma and melanoma. Across multiple phase I/II studies, intratumoral CpG-based regimens induced local immune activation and occasional regression of noninjected lesions, consistent with systemic antitumor activity (35, 36, 82, 85). When CpG-based ISV regimens were combined with RT or ICB, enhanced clinical responses were accompanied by immune correlates, including increased antigen-specific T-cell responses and cytokine production (82, 85). Early clinical evaluation of intratumoral STING agonists revealed evidence of immune activation; however, clinical activity as monotherapy was limited (58). These findings suggest that dose, formulation, and delivery are key determinants of therapeutic activity.

### Cytokines and immune costimulatory agonists

Intratumoral cytokine delivery has been explored as a means to amplify antigen presentation and immune effector cell differentiation while minimizing systemic toxicity. IL-2, IL-12, and GM-CSF have each demonstrated local immune activation in early-phase trials (18, 64). IL-12–based ISV regimens using intratumoral plasmid delivery have shown local and systemic immune activity in melanoma, while localized delivery strategies have been developed to limit the systemic toxicity associated with IL-12. Similarly, intratumoral delivery of costimulatory agonists targeting OX40, CD40, or 4-1BB have demonstrated immune activation, although objective clinical activity has remained limited in early phase studies (65, 66, 86). Collectively, these studies suggest that the clinical activity of cytokines and costimulatory agonists may be enhanced through combination with complementary immunotherapies.

### Biomarkers, endpoints, and trial design considerations

With ISV, it has become clear that traditional endpoints such as ORR and progression-free survival incompletely capture therapeutic impact. Durable response rate, local response assessment, and immune-related response criteria have proven more informative in early-phase studies. Translational endpoints, including TCR repertoire expansion, intratumoral CD8^+^ T-cell density, IFN-I gene signatures and cDC1 abundance, have emerged as critical biomarkers of ISV efficacy. However, these markers are not yet standardized across trials, limiting cross-study comparability. Trial design remains constrained by practical considerations, including tumor accessibility and patient selection. Most studies have preferentially enrolled patients with superficial or oligometastatic disease, underscoring the need for improved delivery approaches to expand the applicability of these treatments (**Figure 3C**) (38, 53, 87).

## Bridging preclinical mechanistic insights to clinical outcomes

Preclinical studies of ISV have established a coherent immunologic framework in which ICD or other forms of immunogenic tumor-cell stress or death, innate immune sensing, and DC-mediated cross-priming cooperate to generate systemic, durable antitumor immunity (62, 63, 79). Clinical trials now provide convergent, though incomplete, evidence that these mechanisms also operate in humans and can be therapeutically exploited when properly engaged (35, 53, 82, 83).

Mechanistically, preclinical models identify IFN-I signaling downstream of cGAS-STING activation as a non-redundant link between local tumor perturbation and adaptive immunity (17, 33). This is reflected clinically, as RT, OVs, and intratumoral innate immune agonists—modalities capable of activating IFN-I programs—have been associated with systemic immune responses and regression of noninjected lesions (35, 53, 82, 83). By contrast, modalities that destroy tumors without sufficient innate immune activation rarely produce abscopal effects in animal models or humans (12).

The central role of Batf3-dependent cDC1s in antigen cross-presentation and CD8^+^ T-cell priming also has strong clinical parallels (7–9, 79). Trials combining ISV with agents that enhance DC recruitment or activation, such as GM-CSF-encoding OVs, intratumoral CpG or CD40 agonists, have shown increased intratumoral CD8^+^ T-cell infiltration, expansion of tumor-reactive TCR clonotypes, and IFN-stimulated gene signatures—biomarkers that closely mirror preclinical requirements for efficacy (54, 65, 66, 82). Their absence in non-responders suggests that insufficient DC licensing remains a major resistance mechanism.

Preclinical evidence for epitope spreading and polyclonal T-cell immunity likewise aligns with clinical findings. In responding patients, ISV has been associated with immune remodeling and responses in noninjected lesions, findings consistent with broader systemic antitumor immunity (37, 38, 54). This feature distinguishes ISV from conventional cancer vaccines and helps explain the durable responses observed with OV-based therapies (53–55).

Despite recent advances, clinical data reveal translational gaps that are not fully captured by preclinical models. Tumor accessibility, delivery heterogeneity, baseline immune contexture, and host immunosuppression introduce complexity that can blunt efficacy in humans. These factors underscore the need for mechanism-informed trial design that incorporates immune stratification, optimized dosing and sequencing, and translational endpoints aligned with ISV biology rather than radiographic response alone.

## Obstacles and future directions

Clinical translation of ISV remains limited by tumor accessibility, uneven intratumoral delivery, and immune suppression within the tumor microenvironment.

Several current efforts to overcome modality-specific limitations are summarized in **Figure 3**. These include optimization of RT dose and fractionation to preserve cGAS–STING–IFN-I signaling while avoiding TREX1-mediated attenuation (33, 34); rational integration of intratumoral innate agonists or locally delivered cytokines (e.g., IL-12, IL-2, GM-CSF) to maximize DC maturation and cross-priming (17–19, 97); and integration of alternative tumor-disrupting or ICD-inducing modalities such as cryoablation, PTT/PDT, and selected chemotherapies (**Figure 1A**). Accumulating clinical experience with RT plus intratumoral innate agonism across lymphoma, sarcoma, Merkel cell carcinoma, and metastatic breast cancer—together with the Flt3L-based ISV sequence—already provides useful guidance for sequencing, dosing, and trial design (35, 75, 83, 90, 91).

Because ISV responses may be delayed yet durable, durable response metrics are particularly appropriate clinical endpoints and should be paired with pharmacodynamic biomarkers, including intratumoral IFN-stimulated gene signatures, spatial and clonal T-cell receptor analyses, and circulating tumor-reactive T cells, to enable mechanistic validation and cross-trial comparability (**Figure 3C**) (87). Advances in intratumoral delivery, including mRNA/LNP formulations, injectable hydrogels, and biodegradable scaffolds, may improve retention at the tumor site and allow more controlled local delivery of immunomodulatory agents. These approaches may enhance local immune activation while limiting systemic exposure and are consistent with the broader strategy of human intratumoral immunotherapy (HIT-IT) (48, 67, 68, 70, 72, 93). Image-guided and endoscopic delivery may expand access to anatomically challenging lesions (**Figure 3C**) (98). OV platforms present additional delivery and engineering challenges. Beyond delivery, genetic engineering of OV platforms may be used to regulate tumor-selective viral replication and therapeutic transgene expression, providing additional control over safety and antitumor activity (49, 100). For systemic administration, cellular carriers are being investigated to shield OVs from neutralizing antibodies and complement and to facilitate tumor-directed delivery (99). A complementary approach is fusogenic engineering to promote syncytium formation and cell-to-cell spread. Because premature fusogenic protein expression can impair viral replication, inducible systems that temporally separate initial viral amplification from subsequent fusion-mediated tumor-cell killing may provide a more controlled strategy (100). Neoadjuvant use of ISV may also support immune priming before surgery (56).

## Conclusion

ISV has evolved from a mechanistic immunology concept into a coherent translational framework that couples tumor-antigen release or availability with innate immune activation and professional antigen presentation, thereby converting a treated tumor into a patient-specific vaccine depot (**Figure 2**). As summarized mechanistically, ICD induces danger signaling and IFN-I programs that activate DCs, particularly cDC1 subsets, enabling efficient antigen cross-presentation and *de novo* priming of polyclonal T-cell responses against a contemporaneous, heterogeneous antigen repertoire that includes both tumor-associated antigens and tumor-specific neoantigens released *in situ* (6, 7, 9, 20).

Across immunocompetent preclinical animal models spanning melanoma, triple-negative breast cancer, colon carcinoma, lymphoma and glioma, this mechanistic cascade can translate into local tumor regression, abscopal-type control of uninjected lesions, and durable immune memory (**Figure 3A**) (62, 63, 69, 78, 88, 89). Effects are most pronounced when antigen release is paired with sufficient innate immune activation, reinforcing the conceptual separation between tumor destruction alone and productive vaccination. Early clinical experience mirrors these principles: intratumoral TLR agonists, often combined with radiotherapy, the sequential Flt3L → low-dose RT → poly-ICLC regimen, intratumoral TLR4 agonism, and the oncolytic HSV-1 platform T-VEC have demonstrated feasibility, immune remodeling within the TME, and, in a small subset of patients, systemic activity at distant lesions (**Figure 3B**) (35, 36, 53, 56, 75, 83, 90, 91).

A potential advantage of intratumoral ISV is the ability to achieve high intralesional drug exposure while limiting systemic exposure, although tolerability depends on the agent, dose, and combination regimen (61, 92, 93). By using endogenous tumor antigens released or presented *in situ*, ISV can also reduce the need for prior antigen identification and ex vivo vaccine manufacture. Functionally, locally primed effector and memory T cells can traffic to noninjected lesions and mediate systemic antitumor responses, including abscopal-type effects (**Figure 2**) (12, 32).

ICB emerges as a rational partner for enhancing clinical responses after ISV (**Figure 3C**). By preventing premature suppression or exhaustion of ISV-primed T cells, ICB can broaden and sustain systemic responses at both injected and noninjected sites (3, 11). Clinically, RT plus intratumoral TLR agonists in lymphoma and cutaneous T-cell lymphoma, the Flt3L-based ISV sequence in indolent lymphoma, and T-VEC used alone or in combination with ipilimumab in melanoma have produced responses in noninjected lesions or improvements in response-based endpoints in selected cohorts (**Figure 3B**) (35, 36, 53, 55, 75, 94, 95). However, a subsequent randomized phase III trial of T-VEC plus pembrolizumab did not significantly improve progression-free or overall survival compared with placebo plus pembrolizumab (84). In parallel, emerging intratumoral nucleic-acid platforms, including nanoplexed poly-IC (BO-112) and local mRNA/LNP strategies such as “Triplet” formulations, further support the principle that potent local innate activation and costimulation can restore or amplify responsiveness to PD-1 blockade, with early clinical evidence for BO-112 and preclinical evidence for mRNA/LNP platforms (70, 78, 96).

Collectively, the evidence reviewed here indicates that the major challenge for ISV is not simply to achieve local tumor control, but to generate systemic and durable antitumor immunity beyond the treated lesion. Across modalities, regression of noninjected lesions, control of established metastatic lesions, prevention of recurrence or metastatic dissemination, and induction of long-term immune memory represent related but distinct outcomes. The reproducibility of these responses depends on treatment dose and sequencing, antigen availability, innate immune activation, delivery efficiency, tumor accessibility, baseline immune contexture, and sustained T-cell function. Standardized reporting of local and systemic responses, together with biomarker-guided trial design and rational combination strategies, will be essential to improve clinical translation and define the settings in which ISV can provide durable disease control.
